# Laser Acupuncture at Large Intestine 4 Compared with Oral Glucose Administration for Pain Prevention in Healthy Term Neonates Undergoing Routine Heel Lance: Study Protocol for an Observer-Blinded, Randomised Controlled Clinical Trial

**DOI:** 10.1155/2018/8406138

**Published:** 2018-03-07

**Authors:** Jasmin Stadler, Alexander Avian, Katrin Posch, Berndt Urlesberger, Wolfgang Raith

**Affiliations:** ^1^Division of Neonatology, Department of Paediatrics and Adolescent Medicine, Medical University of Graz, Auenbruggerplatz 30, 8036 Graz, Austria; ^2^Research Group for Paediatric Traditional Chinese Medicine, TCM Research Centre Graz (Acupuncture Research), Medical University of Graz, Auenbruggerplatz 30, 8036 Graz, Austria; ^3^Institute for Medical Informatics, Statistics and Documentation, Auenbruggerplatz 2, 8036 Graz, Austria

## Abstract

**Background:**

Nonpharmacological strategies have actually become more important in neonatal pain management during routinely applied minor painful procedures. However, commonly used nonpharmacological strategies are inferior to orally administered sweet solutions. Therefore, we will compare laser acupuncture, as a recent nonpharmacological method, with the standard care of oral glucose solution for pain prevention.

**Methods:**

Ninety-five healthy term neonates will be allocated into one of two groups. Before routine heel lance for metabolic screening, one group will receive laser acupuncture at acupuncture point Large Intestine 4 (LI 4) bilaterally for 60 seconds per point (acupuncture group) and the other will receive the standard care with orally administered glucose solution (glucose group). The complete procedure of blood sampling will be recorded on video, excluding the intervention before heel lance. A paediatric nurse, blinded with respect to the allocation, will evaluate these video recordings and determine the Premature Infant Pain Profile (PIPP) for each neonate. Primary outcome will be the mean difference in PIPP scores between groups.

**Discussion:**

This observer-blinded randomised controlled trial has been designed to explore potential advantages of laser acupuncture in the management of neonatal pain because more data are required to provide information about its efficacy and safety.

**Trial Registration:**

This trial is registered with DRKS00010122.

## 1. Background

In recent years, significant progress has occurred, in both the understanding of the physiology of neonatal pain and the efficacy of pharmacological and nonpharmacological interventions [1].

Neonates admitted to hospital are frequently subjected to acute pain, which causes short-term physiological instability and moreover long-term alterations in brain development, behaviour, and stress responses, potentially leading to a greater vulnerability to chronic pain [2–4]. As an example, a frequently used minor painful procedure in clinical routine in neonates is the heel lance for blood sampling.

The actual golden standard for pain prevention during minor painful interventions in preterm and term neonates is the treatment with orally administered sweet solutions. Recent systematic reviews have investigated the effectiveness of sucrose and glucose analgesia in neonates, concluding that both can reduce pain scores and crying duration during minor painful interventions [5, 6]. However, there is still a gap of knowledge regarding the long-term effects of sucrose [5].

Therefore, nonpharmacological strategies have actually become more important in neonatal pain management. Common nonpharmacological therapies are kangaroo care, swaddling, and nonnutritive sucking-related strategies; however, they all are inferior to orally administered sweet solutions but are considered due to their synergistic effects to achieve optimal analgesia [7, 8].

A new nonpharmacological and gentle method for pain prevention in neonates could be laser acupuncture treatment. Laser acupuncture in children and neonates is getting more and more popular, due to the fact that it is a noninvasive and simple-to-perform method. Moreover, the use of laser acupuncture performed within standardised guidelines and in compliance with all safety precautions seems to be safe [9].

Recently, there are only a few trials using acupuncture methods for pain reduction in neonates. Three trials investigated acupuncture in infantile colic and demonstrated significantly a reduction in the total duration of fussing and colicky crying [10–12]. Five other studies investigated acupuncture for pain prevention before heel lance. The first trial demonstrated a significant reduction of pain score PIPP (Premature Infant Pain Profile) [13], one of these showed a reduction of pain score NIPS (Neonatal Infant Pain Scale) and crying duration [14], one illustrated a reduced cry and treatment duration [15], one showed no effect concerning NIPS [16], and the latest trial demonstrated significantly higher PIPP scores only with NESAP (noninvasive stimulation of acupuncture points) and lower PIPP scores with NESAP combined with sucrose solution [17]. The most frequently used acupuncture point in these trials was Large Intestine 4 (Abbreviation: LI 4, in Chinese: He Gu) [10–13].

Therefore, the aim of this trial is to investigate the effect of laser acupuncture at LI 4 for pain prevention in healthy term neonates undergoing minor painful interventions compared to standard care with orally administrated glucose solution.

## 2. Methods

### 2.1. Design

This study is designed as a single centre, observer-blinded, noninferiority, randomised, clinical controlled trial with two study arms, started on 14 March 2016, and collected data until the end of 2016. This trial is performed at the Division of Neonatology, Department of Paediatrics and Adolescent Medicine, Medical University of Graz, Austria, Europe.

This trial is registered in German Clinical Trials Register with the registry number DRKS00010122 on 2 March 2016.

### 2.2. Participant Enrolment and Eligibility Criteria

We will enrol and include healthy term neonates (>37 weeks' gestational age) without clinical and/or congenital complications, after spontaneous delivery or Caesarean section, receiving heel lance for metabolic screening between 2nd and 5th day postnatally and with written informed consent signed by the parents.

The participants will not be enrolled or be excluded, if there will be lack of written informed consent or current pain therapy in the neonate and/or in the breastfeeding mother. We will not enrol preterm neonates (<37 weeks' gestational age), neonates with clinical and/or congenital diseases, and neonates who are not receiving heel lance for metabolic screening.

Parents of neonates matching the inclusion criteria will be informed about the trial on the first days after delivery. To guarantee achieving adequate participant enrolment, the nursing staff at Department of Obstetrics and Gynaecology, Medical University of Graz, will be informed about the course of the study, and informational conversation with parents will be held daily. After requiring a signed written informed consent, the participant data will be collected and the heel lance will be performed from a medical doctor at the Division of Neonatology in a special treatment room.

### 2.3. Participants

This study will include 95 healthy term neonates during their hospitalisation after delivery. Using the Randomizer for Clinical Trials tool (https://www.randomizer.at/) developed at the Medical University of Graz, neonates will be allocated into one of two groups. One group is the intervention group and will receive laser acupuncture at acupuncture point LI 4 bilaterally (acupuncture group = AG). The other group is the active control group and will receive standard care treatment with orally administered glucose solution (glucose group = GG).

### 2.4. Intervention

Before the procedure starts, participants will be randomised in AG or in GG. Neonates in both groups will be monitored with a pulse oximeter (IntelliVue X2, Philips Medicin System, Boeblingen GmbH, Boeblingen, Germany) across the right wrist to observe heart rate and peripheral oxygen saturation during the intervention and the blood sampling. In addition, neonates in both groups will wear an eye protector (Natus Biliband Eye Protector, Natus Medical, San Carlos, California) during the treatment with laser acupuncture or oral glucose solution, following the same treatment protocol so that all participants will be manipulated equally. The eye protector will be removed 30 seconds before the heel lance.

*AG*. Participants will receive laser acupuncture at LI 4 bilaterally, 60 seconds at the left point and 60 seconds at the right point, in total 120 seconds. 30 seconds until heel lance are left.

*GG*. Neonates in this group will receive orally administered glucose solution for 30 seconds and two minutes waiting period until heel lance.

The intervention in both groups will take in total 150 seconds. Afterwards, the standardised process of blood sampling with heel lance will be carried out.

The complete procedure of blood sampling will be recorded on video, excluding the intervention with laser acupuncture or oral glucose solution. The video will start 15 seconds before heel lance and will record until blood sampling is completed. These video recordings will be analysed by a certified paediatric nurse, who will determine the PIPP for each neonate, blinded to group allocation. To improve the adherence to the study protocol a medical doctor is following written standards of procedure (SOP).

**Figure 1:**
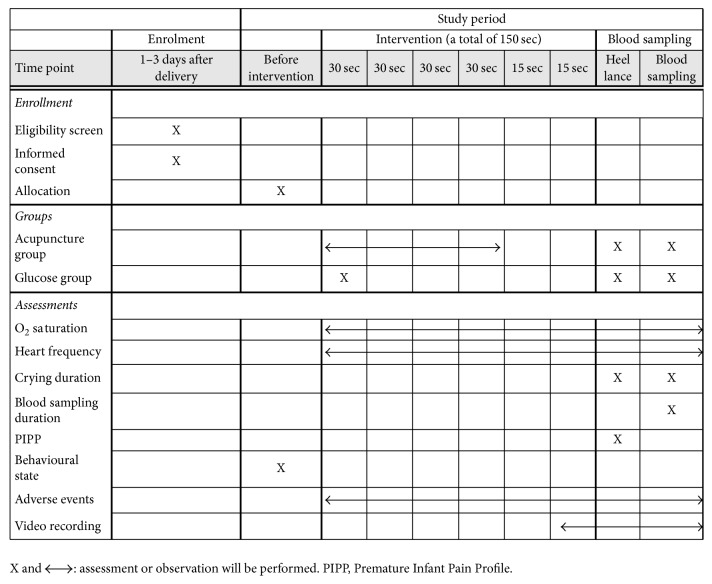
Time schedule of enrolment and assessments.

**Figure 2:**
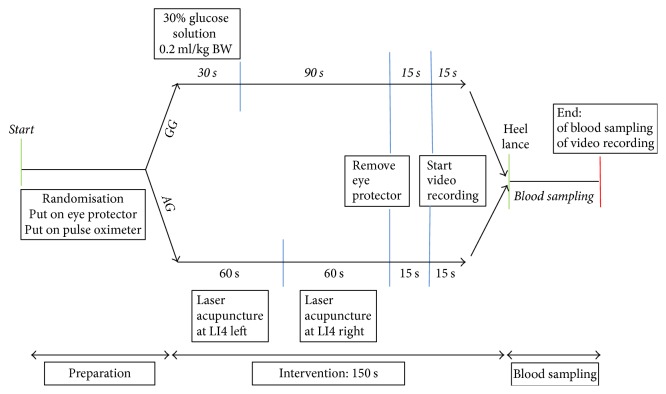
Time chart of study course. AG, acupuncture group; GG, glucose group; LI 4, Large Intestine 4; s, seconds.

Figure 1 shows the time schedule of enrolment and assessments and Figure 2 gives information about the study course.

### 2.5. Outcome Measurement

The primary outcome is the mean difference in PIPP score between AG and GG.

Secondary outcomes will be mean differences in heart rate change from baseline, in oxygen saturation change from baseline, and in crying time, as well as any adverse events during intervention.

Explorative outcome parameters will be general behavioural state (sleeping, awake, agitated, or crying) of the neonate before intervention, the last breastfeeding time point before intervention, and the number of heel lances performed that will be notated. The duration of blood sampling will be recorded in seconds.

### 2.6. Minor Painful Intervention

In this study, all participants will receive a heel lance by automatic lancet (gentleheel®, Alleset Healthcare Solutions B.V., Netherlands) as a routinely used painful minor intervention for blood sampling for metabolic screening [18]. The National Austria Newborn Screening Program started in 1966 and identifies inherited metabolic and endocrine disorders, which every newborn in Austria receives between 2nd and 5th day of life [19].

Before the heel lance, the skin of the newborn will be prepared using a disinfectant (kodan® forte farblos, Schülke & Mayr GmbH, Norderstedt, Germany) and cellulose swabs (Profümed®, Profümed GmbH, Grimmenstein, Austria).

### 2.7. Premature Infant Pain Profile (PIPP)

The PIPP score is a possibility to evaluate acute pain intensity and is largely used and validated for preterm and term neonates during a painful intervention [20, 21]. Ballantyne et al. demonstrated a good construct validity, differentiating pain from nonpain and baseline events (*F* = 48, *p* = 0.0001), as well as interrater reliability (reliability coefficients of 0.93–0.96) and intrarater reliability (reliability coefficients at 0.94–0.98) for pain assessment in preterm and term neonates [22].

The following indicators will be evaluated: (1) gestational age, (2) behavioural state before painful stimulus, (3) change in heart rate during painful stimulus, (4) change in oxygen saturation during painful stimulus, (5) brow bulge during painful stimulus, (6) eye squeeze during painful stimulus, and (7) nasolabial furrow during painful stimulus.

In each category 0 to 3 points can be allocated. Conversely, point 1, gestational age, and point 2, behavioural state before painful stimulus, have a reversed score (3, 2, 1, and 0) accounting for physiological differences related to prematurity.

The maximum PIPP score for preterm < 28 weeks' gestational age is 21 points and 18 points for term neonates. Accordingly, the number of achieved points on the PIPP score is directly proportional to the neonate's pain intensity during the painful intervention.

### 2.8. Oral Sweet Solution

The oral glucose solution will be a 30% glucose solution (Glux® from Pharma Stulln, Germany), which each patient in the GG will receive within 30 seconds, 150 seconds before the intervention. The application and dosage of the oral glucose solution during minor painful interventions are based on the Austrian interdisciplinary guidelines for perioperative pain management [23]. A dosage of 0.2 ml/kg body weight is recommended.

### 2.9. The Acupuncture Point

LI 4 is called in Chinese “He Gu,” which means “Union Valley.” LI 4 is located on the dorsal side of the hand in the middle between 1st and 2nd metacarpal bones, in direction to the middle of the 2nd metacarpal bone. This acupuncture point is one of the most frequently used points in acupuncture and is the main point against any kind of pain.

The acupuncture point is described following the standard of international acupuncture nomenclature [24]. In addition, the acupuncture treatment is described according to the established guidelines of STandards for Reporting Interventions in Clinical Trials of Acupuncture (STRICTA) [25].

### 2.10. Technical Parameters of Laser Acupuncture

The AG will receive laser acupuncture using LABpen MED 10 (Behounek, Graz, Austria). This Laser is a continuous wave semiconductor GaAs (Gallium Arsenide) laser with wavelength of 675 nm and output power of 10 mW, with diameter 1.5 mm [26].

The body acupuncture point LI 4 will be treated for 60 seconds per point, in total 120 seconds (=0.6 J/point, which corresponds to an energy dosage of 34 J/cm^2^) [27].

### 2.11. Safety Precautions during Laser Acupuncture

The new classification for low-level lasers according to the European Norm (EN 60825-1) is 3R, which means that radiation can cause serious eye damage [26].

Therefore, the use of protective glasses to avoid retinal damage is necessary. The acupuncturist and any other person in the treatment room will wear specific protective glasses. All included neonates will wear an eye protector (Natus Biliband Eye Protector, Natus Medical, San Carlos, California), which is routinely used in neonates during phototherapy for neonatal jaundice [28], on the one hand to avoid eye damage in neonates who receive laser acupuncture and on the other hand to manipulate all participants in both groups equally. The eye protector will be removed 30 seconds before heel lance.

The Department of Technical and Organisational Safety of the Medical University of Graz has examined and approved the effectiveness of this eye protector and application of the low-level laser.

### 2.12. Allocation and Randomisation

Neonates will be randomly allocated to laser acupuncture or orally administered glucose solution in a 1 : 1 ratio using an online software program for clinical trials (https://www.randomizer.at) developed at the Medical University of Graz. Just before the intervention, the included participant's code and sex will be typed in by the principal investigator and the program will show the allocation of this participant. Therefore the principal investigator will only know the allocation of this and the previous included neonates; the allocation of any upcoming participants will not be visible. Only the biometrician knows the used randomisation algorithm. If a neonate has to be excluded after randomisation, the reason for revision of randomisation will be documented within the program. This revision can only be done by the biometrician. Randomisation will not be stratified for any factor.

### 2.13. Blinding

The blinding will be “observer blinded,” due to the fact that the video recordings will be analysed by a skilled paediatric nurse, who will know neither the participant nor the intervention. To guarantee blinding, these video recordings will show only 15 seconds before the heel lance and the blood sampling. The interventions with laser acupuncture or oral glucose solution will not be recorded.

### 2.14. Data Management

Pseudonymous data will be recorded first on a Case Report Form and then on an Excel Sheet (Microsoft Excel 2010, Microsoft Corporation, Redmond, Washington, USA) for statistical analysis including data from the neonate, the mother, and the delivery, as well as information recorded during the intervention. Outcome parameters will be collected before and during intervention (e.g., heart frequency, oxygen saturation) and on the basis of video recordings of the participants (e.g., crying duration, PIPP). Adverse events will be collected and reported during the intervention and supervised by a paediatrician. However, from preliminary trials, little to no risk for adverse events is expected for the included neonates. If there will be suspicion of adverse events or harm in most cases, discontinuing or modifying allocated interventions for given trial participants will be reviewed. Furthermore, interim analyses will not be performed.

Data will be stored on restricted-access files at the Division of Neonatology, except for the study contributors.

### 2.15. Sample Size

In a group of neonates receiving laser acupuncture, Gottschling et al. reported a pain reaction following heel lance of 6.4 ± 1.9 in PIPP score [13]. Assuming a SD (standard deviation) of 1.9 in pain scores of neonates undergoing heel lance, a sample size of 95 neonates will have a power of 80% (one-sided *t*-test) to reject the null hypothesis that the laser acupuncture and oral glucose administration are not equivalent (the difference in means, *μ*AG  −  *μ*GG, is 1.0 or farther from zero in the same direction) in favour of the alternative hypothesis that the means of the two groups are equivalent, assuming that the expected difference in means is 0. Assuming a minimal dropout of *n* = 5 neonates, we expect to include 95 participants in total.

### 2.16. Statistical Analysis

We will compare the PIPP scores of both groups using intention-to-treat analysis (ITT). Furthermore, per-protocol (PP) analysis will be performed, since ITT analysis increases the chance of finding noninferiority (increase of type I error) [29]. Results of ITT and PP analysis will be compared.

First baseline characteristics of both groups (gestational age, sex, APGAR score, birth weight, birth size, head circumference, birth mode, and day of life at the time of heel lance) will be compared using *t*-test or Mann–Whitney *U* test for continuous variables and chi-square test or Fisher's exact test as appropriate. For differences in these characteristics, further analysis will be adjusted.

Laser acupuncture will be considered noninferior to oral glucose administration, if the upper boundary of the two-sided 95% confidence interval (CI) for the mean difference between laser acupuncture and oral glucose administration will be less than the margin, *δ* = 1. If data will not be normally distributed, nonparametric tests will be applied.

For ITT analysis, missing primary outcome measures will be imputed by using multiple imputations. For PP analysis, no imputations will be performed. Secondary outcomes will be compared using *t*-test or Mann–Whitney *U* test for continuous variables and chi-square test or Fisher's exact test as appropriate. Since interventions will be of short duration and no risks are associated with the intervention, no interim analysis will be performed.

The used statistic programs for statistical analysis will be IBM SPSS Statistics (Statistical Package for the Social Sciences) and SAS (state-of-the-art statistical analysis software).

The primary outcome measure is the PIPP score as defined above.

### 2.17. Ethics and Dissemination

Participants in this trial will be protected against invasion of privacy. Therefore, the collected data and video recordings will be pseudonymous, securely stored at Division of Neonatology. This study has been approved by the Ethic Committee Graz (28-217 ex 15/16) and will be conducted with respect to the individual participants according to the Declaration of Helsinki and the Guidelines for Good Clinical Practice. The study protocol is reported in accordance with the Standard Protocol Items: Recommendations for Interventional Trials (SPIRIT Checklist 2013) [30].

The results of the present trial will be published in a peer-reviewed journal and presented at relevant conferences.

## 3. Discussion

The present observer-blinded, randomised, controlled trial has been designed to explore potential advantages of laser acupuncture in the management of neonates' pain. Due to the fact that recent evidence proved the necessity to improve healthcare and pain management in neonates, it is important to investigate new methods for pain management to provide pain associated adverse effects. Therefore, it is also important to assess pain with a validated and frequently used pain score, for example, the PIPP score, which is consistently used in clinical neonatal settings and research context providing a reliable tool for neonatal pain assessment [20–22].

In preliminary studies about acupuncture for pain relief in neonates, different results were reported. Significant pain reduction has been demonstrated in two studies, one with laser acupuncture with 30 mW at LI 4 and Shen Men and the other with light needling at Yin Tang [13, 14]. One study with acupressure showed equal pain scores and one higher pain scores in neonates treated with laser acupuncture with 10 mW at Yin Tang [15, 16]. The latest study showed significant pain reduction with NESAP combined with oral sucrose solution [17].

Due to heterogeneity across studies in regard to acupuncture modality and used acupuncture points, it is difficult to compare the results and to give clear recommendation.

We will discuss our study with the results in regard to the effectiveness of laser acupuncture and to the acupuncture LI 4 and also in regard to our control group with oral glucose solution.

One limitation of our study is the absence of a placebo control group. However, according to recent recommendations, although the placebo or sham effects of acupuncture can be estimated using experimental trials in adults, this is not the case for clinical trial with neonates suffering from pain [31].

## 4. Conclusion

Based on the heterogeneity across preliminary studies about acupuncture for pain relief in neonates, clear recommendations are missing and, therefore, it is important to add new evidence. To date, no trial has been carried out comparing laser acupuncture at LI 4 with oral glucose solution in a noninferiority setting to quantify the benefits.

Including the well-proven specificity of acupuncture at LI 4 for pain relief, we hypothesize that laser acupuncture at LI 4 will reduce pain and distress associated with minor painful procedures in healthy term neonates equally to orally administrated glucose solution.

## Abbreviations

AG: Acupuncture group
BW: Body weight
CI: Confidence interval
cm: Centimetre
e.g.,: Exempli gratia
GaAs: Gallium arsenide
GG: Glucose group
ITT: Intention-to-treat analysis
J: Joule
kg: Kilogram
LI 4: Intestine 4
mm: Millimetre
mL: Millilitre
mW: Milliwatt
NIPS: Neonatal Infant Pain Scale
PIPP: Premature Infantile Pain Profile
PP: Per-protocol analysis
SAS: State-of-the-art statistical analysis software
Sec: Seconds
SD: Standard deviation
SPIRIT: Standard Protocol Items: Recommendations for Interventional Trials
SPSS: Statistical Package for the Social Sciences
SOP: Standards of procedure
ST: STandards for Reporting Interventions in Clinical Trials of Acupuncture.
